# Distinct synaptic pools of DAPK1 differentially regulate activity-dependent synaptic CaMKII accumulation

**DOI:** 10.1016/j.isci.2023.106723

**Published:** 2023-04-23

**Authors:** Jonathan E. Tullis, K. Ulrich Bayer

**Affiliations:** 1 Department of Pharmacology, University of Colorado Anschutz Medical Campus, Aurora, CO 80045, USA; 2 Program in Neuroscience, University of Colorado Anschutz Medical Campus, Aurora, CO 80045, USA

**Keywords:** Molecular neuroscience, Cellular neuroscience

## Abstract

The death-associated protein kinase 1 (DAPK1) regulates the synaptic movement of the Ca^2+^/calmodulin (CaM)-dependent protein kinase II (CaMKII). Synaptic CaMKII accumulation is mediated via binding to the NMDA-receptor subunit GluN2B and is required for long-term potentiation (LTP). By contrast, long-term depression (LTD) instead requires specific suppression of this movement, which is mediated by competitive DAPK1 binding to GluN2B. We find here that DAPK1 localizes to synapses via two distinct mechanisms: basal localization requires F-actin, but retention of DAPK1 at synapses during LTD requires an additional binding mode, likely to GluN2B. While F-actin binding mediates DAPK1 enrichment at synapses, it is not sufficient to suppress synaptic CaMKII movement. However, it is a prerequisite that enables the additional LTD-specific binding mode of DAPK1, which in turn mediates suppression of the CaMKII movement. Thus, both modes of synaptic DAPK1 localization work together to regulate synaptic CaMKII localization and thereby synaptic plasticity.

## Highlights

• Basal synaptic DAPK1 localization requires F-actin
• cLTD stimuli promote actin-independent DAPK1 synaptic retention
• This indicates two distinct modes of synaptic DAPK1 localization
• Both DAPK1 modes act together to suppress CaMKII movement during LTD

## Graphical abstract

**Figure:**
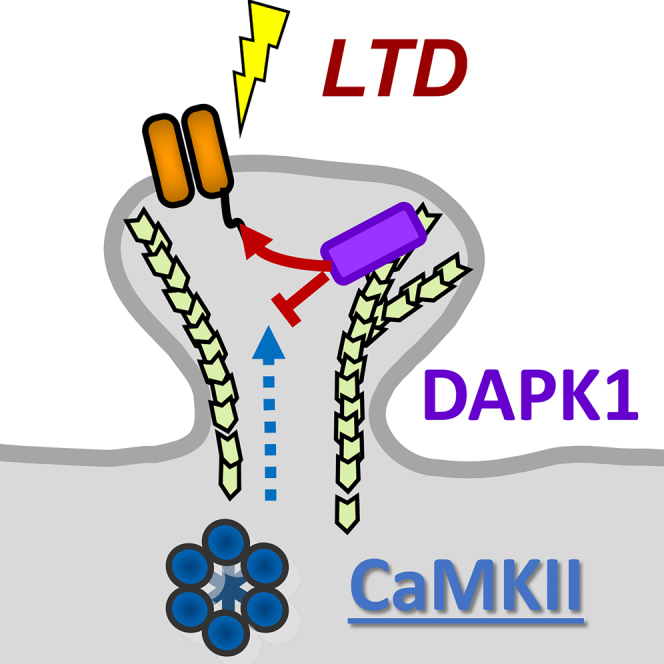

## Introduction

Learning, memory, and cognition are thought to require hippocampal long-term potentiation (LTP) and long-term depression (LTD), two opposing forms of synaptic plasticity.^1^^,^^2^^,^^3^ Both LTP and LTD require the Ca^2+^/calmodulin (CaM)-dependent protein kinase II (CaMKII), with the direction of plasticity determined by its differential synaptic targeting^4^: LTP requires further post-synaptic accumulation of CaMKII at excitatory synapses^5^^,^^6^; by contrast, LTD specifically requires suppression of this movement.^7^^,^^8^

The synaptic accumulation of CaMKII in response to LTP stimuli is mediated by regulated binding to the NMDA-type glutamate receptor (NMDAR) subunit GluN2B.^6^^,^^9^^,^^10^^,^^11^ By contrast, in response to LTD stimuli, this GluN2B binding and the synaptic accumulation of CaMKII is suppressed by two specific mechanisms: autophosphorylation of CaMKII T305/306^8^ and activation of death-associated protein kinase 1 (DAPK1).^7^ The autophosphorylation at T305/306 directly reduces CaMKII binding to GluN2B,^12^ and DAPK1, another member of the CaM kinase family,^4^ binds to the cytoplasmic C-terminus of GluN2B near S1303, at a site overlapping the CaMKII-binding site.^7^^,^^13^ These two suppression mechanisms are required simultaneously, as disabling either one of them is sufficient to allow synaptic CaMKII accumulation even after LTD stimuli, even with the other suppression mechanism intact.^7^^,^^8^ The suppression by DAPK1 requires its successful competition with CaMKII for binding to GluN2B after LTD but not LTP stimuli, which in turn requires activation of DAPK1.^7^ The importance of DAPK1 activation for suppressing CaMKII binding was further highlighted by a pharmacogenetic approach: LTD was blocked by DAPK1 inhibition, but only in hippocampal slices from wild-type mice and not in slices from mice with a GluN2B mutation that already directly suppressed CaMKII binding.^7^ Synaptic targeting of DAPK1 is thought be mediated by its CaMKII-competitive GluN2B binding;^7^^,^^13^^,^^14^ however, DAPK1 also contains a binding site for F-actin, a cytoskeletal scaffold that is basally enriched in the dendritic spines that form the post-synaptic compartments of excitatory synapses. We decided to investigate the contribution of F-actin to the post-synaptic targeting of DAPK1 and CaMKII to dendritic spine synapses in hippocampal neurons, both before and after chemical stimuli that induce LTP or LTD (cLTP or cLTD).

Here, we show that F-actin was required for the extremely high synaptic enrichment of DAPK1 under basal conditions. After stimulation, the F-actin binding by itself was not sufficient to suppress synaptic CaMKII accumulation. However, the basal F-actin binding was required for an LTD-induced transition of DAPK1 to a different, likely GluN2B-bound state. This LTD-dependent DAPK1 state was no longer dependent on F-actin for synaptic targeting and was essential for suppressing synaptic CaMKII accumulation. Thus, our results reveal two distinct synaptic pools of DAPK1 and their distinct functions in regulating the differential synaptic accumulation of CaMKII during LTP versus LTD.

## Results

### DAPK1 basal synaptic localization is dependent on F-actin

**Figure 1:**
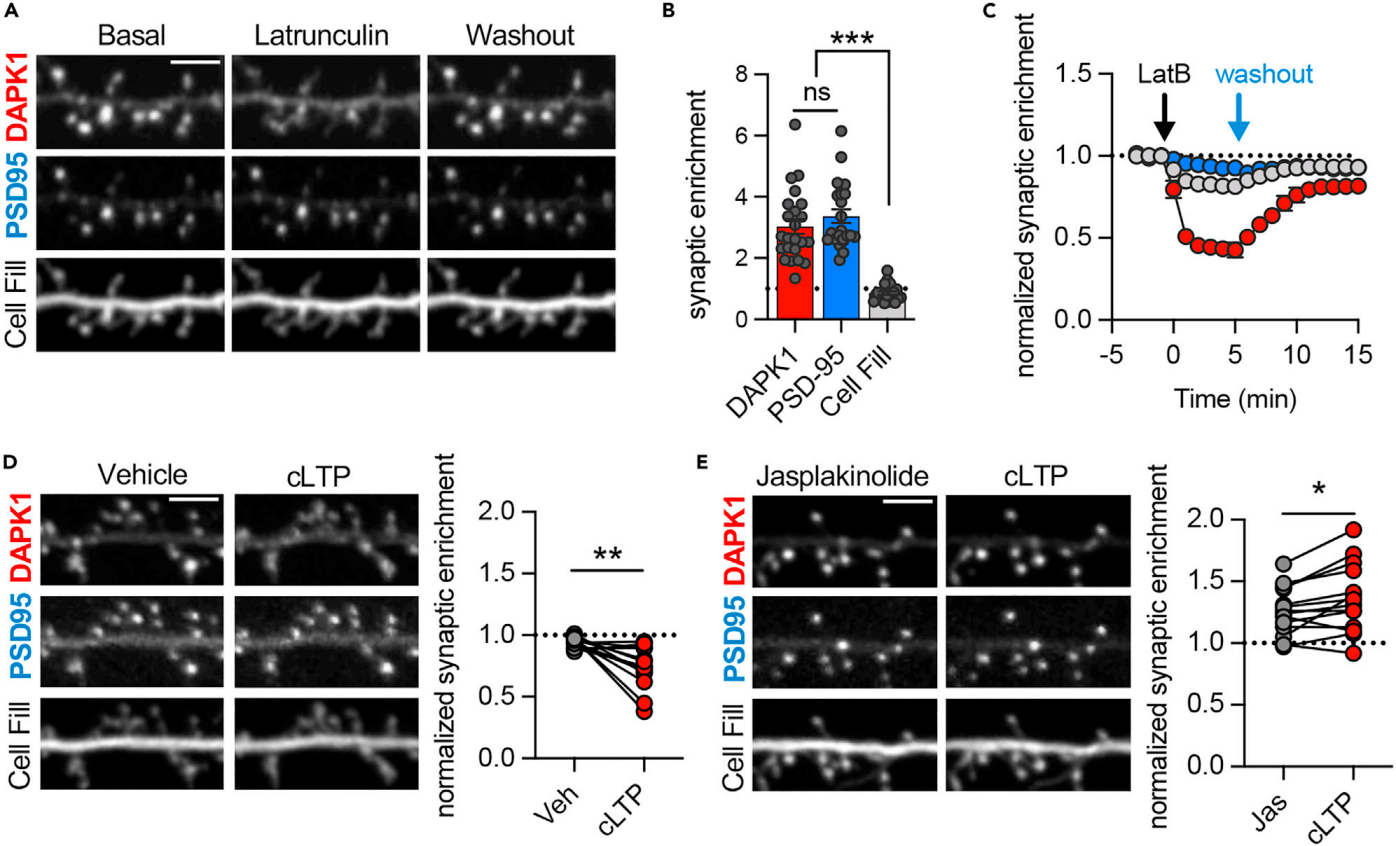
DAPK1 basal synaptic enrichment requires F-actin, and stabilization of F-actin during cLTP blocks DAPK1 synaptic removal Scale bars, 5 μm. (A) Representative images of mCh-DAPK1, mTq-PSD95 intrabody, and mCitrine cell fill. DAPK1 synaptic enrichment values were measured over time following treatment with Latrunculin B (2.5 μM) and washout after aCSF. (B) Basal synaptic enrichment values of DAPK1, PSD95, and cell fill were compared (n = 24 neurons; ∗∗∗p < 0.001; RM one-way ANOVA with Tukey’s multiple comparisons test). Individual data points are shown; the bar graphs represent mean ± SEM. (C) Time course of DAPK1, PSD95, and cell fill synaptic enrichment values after treatment with Latrunculin B for 5 min followed by a washout with aCSF (n = 8 neurons). Data are represented as mean ± SEM. (D and E) (D) cLTP stimulus (100 μM glutamate/10 μM glycine, 1 min) promoted the reduction of DAPK1 synaptic enrichment (n = 13 neurons; ∗∗p < 0.01; paired two-tailed t-test), but (E) stabilization of F-actin with jasplakinolide (1 μM, 10 min) instead blocked DAPK1 synaptic dispersal after cLTP (n = 14 neurons; ∗p < 0.05; paired two-tailed t-test).

In order to monitor the basal localization of DAPK1 as well as its movement upon stimulation, we imaged mCherry-DAPK1 in live cultured hippocampal neurons (Figure 1A). Synaptic localization was determined by the co-localization with PSD95, which was detected with an mTurquoise-labeled intrabody.^15^^,^^16^ Additionally, changes in spine size were monitored using mCitrine as a cell fill. Under basal conditions, DAPK1 was highly enriched at dendritic spine synapses, nearly to the same extent as PSD95 (Figures 1A and 1B). To determine the contribution of F-actin to this synaptic enrichment, we tested the effect of latrunculin B (2.5 μM), a potent inhibitor of F-actin polymerization that leads to rapid overall depolymerization^17^^,^^18^ (Figure S1). Almost immediately after application, latrunculin led to the dispersal of DAPK1 from synapses, becoming unenriched at synapses within 5 min (Figures 1A and 1C). By contrast, spine size and PSD-95 puncta were not at all or only mildly diminished (Figures 1A and 1C), indicating synapses were still intact. Upon washout of latrunculin, DAPK1 re-accumulated at synapses within 10 min (Figures 1A and 1C).

### Removal of DAPK1 from synapses by cLTP is blocked by stabilizing F-actin

DAPK1 was removed from synapses after depolymerizing F-actin (Figure 1C), an effect also seen after chemical LTP stimuli (cLTP; 100 μM glutamate, 10 μM glycine for 1 min) (Figure 1D).^7^^,^^19^ Thus, as LTP involves dynamic F-actin remodeling,^20^^,^^21^^,^^22^ which requires transient F-actin depolymerization, we decided to test if F-actin depolymerization is required for the cLTP-induced DAPK1 dispersal. Stabilizing F-actin with jasplakinolide (1 μM, 10 min) led to a mild increase in basal DAPK1 synaptic enrichment (Figure S2), further demonstrating the role of F-actin in the basal synaptic localization of DAPK1. More importantly, jasplakinolide completely blocked the removal of DAPK1 in response to cLTP stimuli (Figure 1E), indicating that transient F-actin depolymerization after LTP stimuli is indeed required for the synaptic dispersal of DAPK1.

### LTD stimuli make synaptic DAPK1 localization largely independent of F-actin

**Figure 2:**
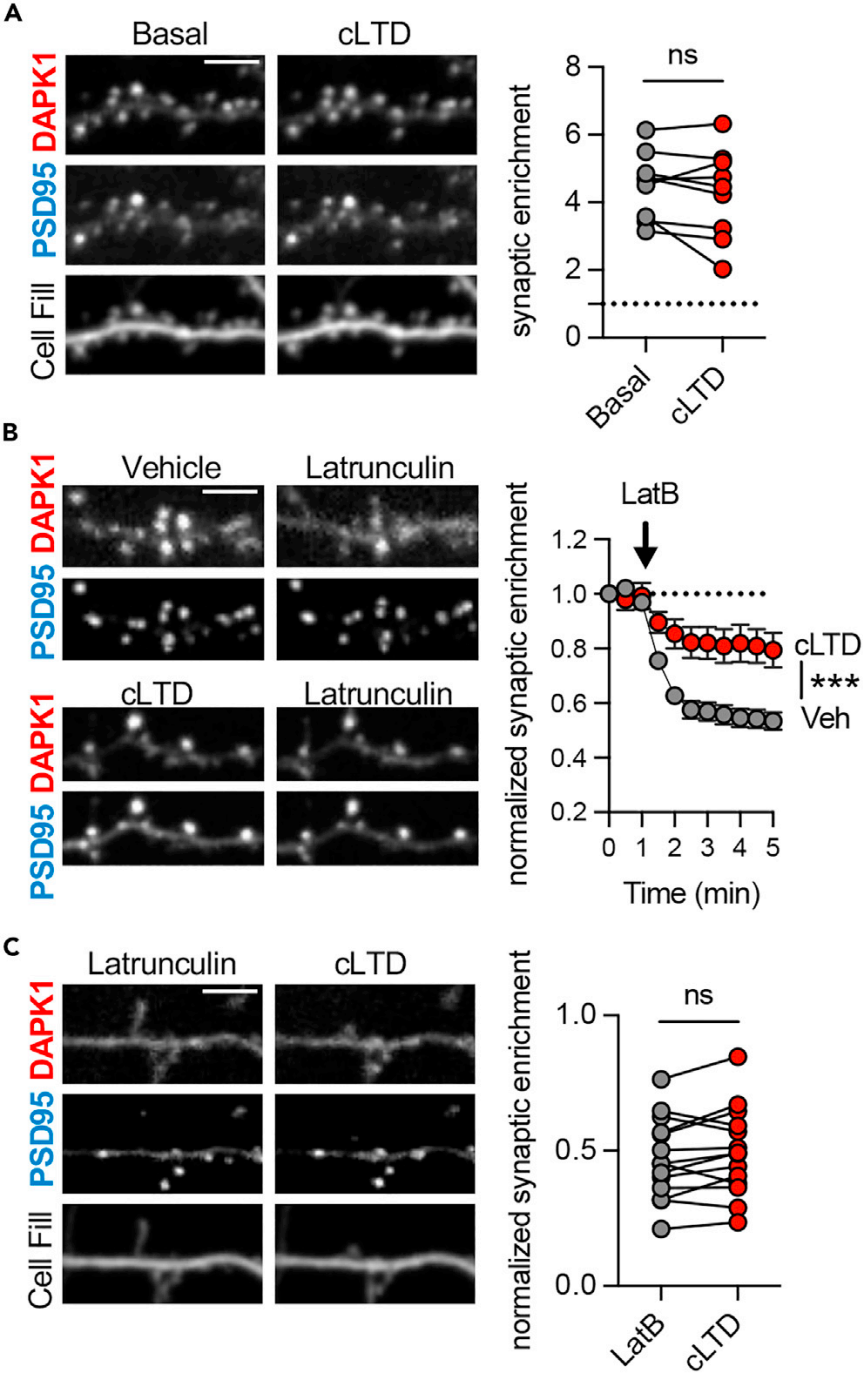
DAPK1 localization to synapses via F-actin is required for initiation of cLTD-specific DAPK1 synaptic pool Scale bars, 5 μm. (A) cLTD stimulation (30 μM NMDA/10 μM CNQX/10 μM glycine, 1 min) did not affect DAPK1 synaptic enrichment (n = 9 neurons; paired two-tailed t-test). (B) cLTD stimulation 1 min prior to application of Latrunculin B impaired the Latrunculin-induced removal of DAPK1 from synapses (n = 15, 16 neurons; ∗∗∗p < 0.001; two-way RM ANOVA). Data are represented as mean ± SEM. (C) cLTD stimulation following Latrunculin-induced removal of DAPK1 did not result in synaptic re-accumulation of DAPK1 (n = 14 neurons; paired two-tailed t-test).

If basal synaptic DAPK1 localization requires F-actin and its dispersal from synapses after cLTP requires F-actin depolymerization, then why does DAPK1 not disperse after chemical LTD stimuli (cLTD; 30 μM NMDA, 10 μM CNQX, 10 μM glycine for 1 min; Figure 2A),^7^^,^^8^ which cause a much more extensive and prolonged depolymerization of F-actin^23^^,^^24^? In order to address this question, we monitored the effect of cLTD stimuli on DAPK1 localization either before or after inducing F-actin depolymerization by latrunculin. Interestingly, when cLTD stimuli were applied prior to latrunculin, the removal of DAPK1 from synapses that is caused by depolymerization of F-actin was significantly reduced (Figure 2B), indicating a transition from an F-actin-bound state, to an LTD-specific state that is no longer dependent on F-actin for its synaptic localization. We then asked whether cLTD stimuli after latrunculin treatment would lead to synaptic re-accumulation of DAPK1 after its prior dispersal that was caused by the F-actin depolymerization. In the continued presence of latrunculin, cLTD stimuli did not promote the synaptic re-accumulation of DAPK1 (Figure 2C). These results indicate that the basal synaptic localization of DAPK1 via F-actin is essential for the subsequent LTD-induced formation of a distinct synaptic DAPK1 pool that is retained at synapses independently of F-actin.

### Synaptic removal of DAPK1 by latrunculin B enables CaMKII accumulation at synapses in response to cLTD stimuli

**Figure 3:**
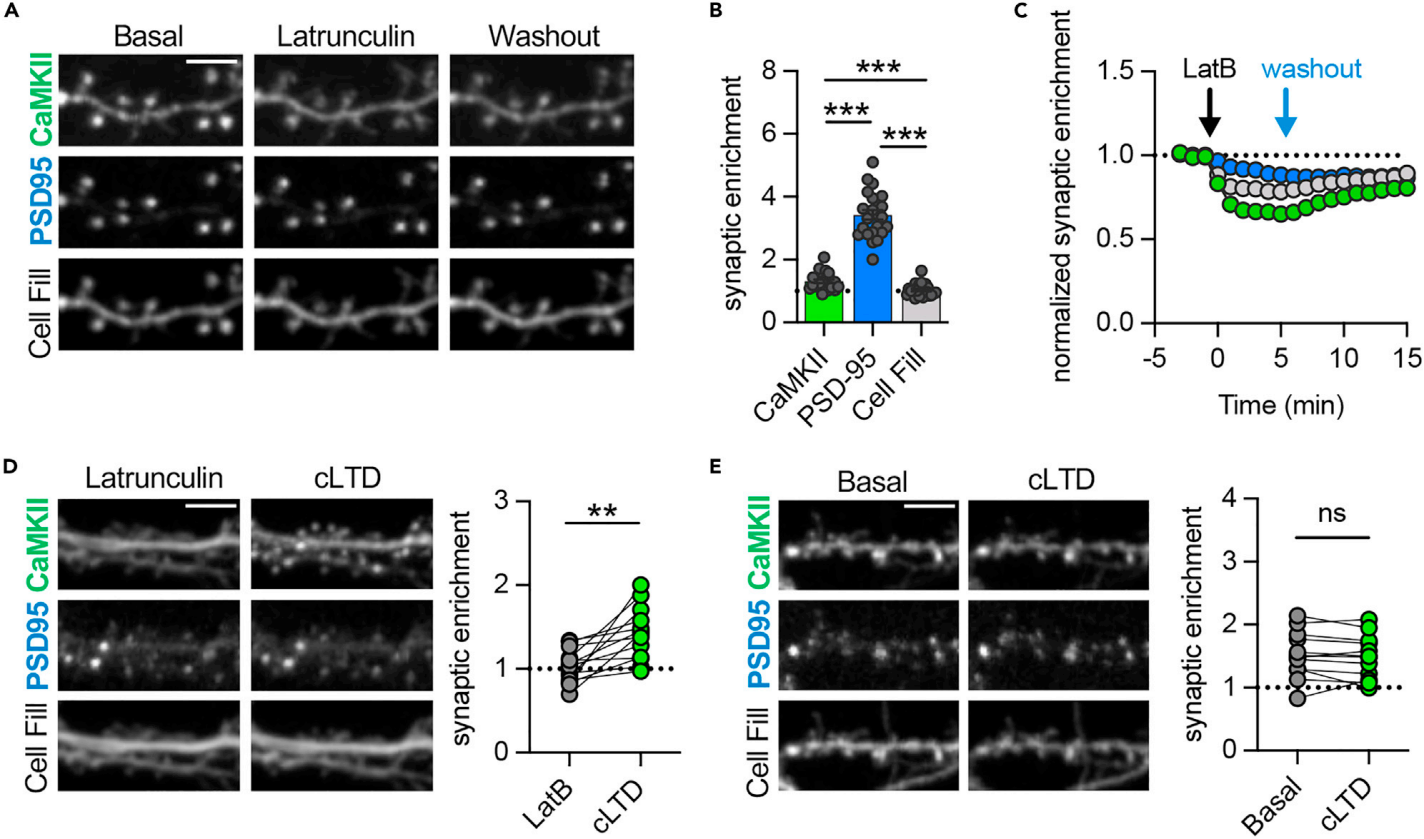
F-actin targeting of DAPK1 is required to suppress synaptic CaMKII accumulation during LTD Scale bars, 5 μm. (A) Representative images of mCh- CaMKII, mTq-PSD95 intrabody, and mCitrine cell fill. CaMKII synaptic enrichment values were measured over time following treatment with Latrunculin B (2.5 μM) and washout after aCSF. (B) Basal synaptic enrichment values of CaMKII, PSD95, and cell fill were compared (n = 23 neurons; ∗∗∗p < 0.001; RM one-way ANOVA with Tukey’s multiple comparisons test). Individual data points are shown; the bar graphs represent mean ± SEM. (C) Time course of CaMKII, PSD95, and cell fill synaptic enrichment values after treatment with Latrunculin B for 5 min followed by a washout with aCSF (n = 8 neurons). Data are represented as mean ± SEM. (D) cLTD stimulation following Latrunculin treatment resulted in the F-actin-independent synaptic accumulation of CaMKII (Latrunculin included in washout after cLTD, n = 13 neurons; ∗∗p < 0.01; two-tailed t-test). (E) cLTD stimulation did not affect CaMKII synaptic enrichment (n = 12 neurons; paired two-tailed t-test).

Under basal conditions, CaMKII is enriched at excitatory synapses (Figure 3A),^4^^,^^25^ but to a much lesser extent than PSD95 or DAPK1 (Figure 3B; compare also Figures 1A and 1B). In response to cLTD stimuli, CaMKII neither disperses nor accumulates,^7^^,^^8^^,^^26^ with the accumulation specifically blocked by competitive GluN2B binding of DAPK1, which is retained at synapses after cLTD stimuli and dispersed after cLTP stimuli.^7^^,^^19^ Thus, we decided to test if synaptic DAPK1 dispersal induced by latrunculin similarly enables synaptic CaMKII accumulation in response to cLTD stimuli. As done in the previous experiments for DAPK1, localization of mCherry-CaMKII was monitored in live hippocampal neurons. The latrunculin treatment mildly reduced the synaptic localization of CaMKII, with a partial return to basal localization after washout of latrunculin (Figure 3C). This synaptic CaMKII dispersal was much less extensive compared to DAPK1, but still slightly exceeded the parallel reduction in spine density (Figure 3C). Most importantly, when latrunculin treatment was maintained to keep DAPK1 dispersed, additional cLTD stimuli now allowed significant re-accumulation of CaMKII at synapses (Figure 3D), similar as described after DAPK1 inhibition or knockout.^7^^,^^8^ Without latrunculin, the cLTD stimuli did not induce any CaMKII movement to excitatory synapses (Figure 3E), as expected. This indicates that the basal synaptic targeting of DAPK1 by F-actin is required for suppressing synaptic CaMKII accumulation in response to LTD stimuli.

### CaMKII is not required to suppress synaptic DAPK1 accumulation during LTD after latrunculin B treatment

While cLTD stimuli retained DAPK1 at synapses during subsequent latrunculin treatment (see Figure 2B), latrunculin treatment did not allow DAPK1 re-accumulation at synapses after cLTD stimuli (see Figure 2C). As latrunculin treatment enabled CaMKII accumulation at excitatory synapses even in response to cLTD stimuli, this suggested that DAPK1 re-accumulation competitive maybe suppressed by competitive binding of CaMKII to GluN2B under these conditions. However, the same failure of synaptic DAPK1 re-accumulation was seen also in hippocampal cultures from CaMKIIα knockout mice (Figure S3). Thus, CaMKIIα, the major CaMKII isoform in the brain, is not required to suppress the DAPK1 re-accumulation; instead, the results indicate that the basal DAPK1 interaction with F-actin is required to enable the transition to a DAPK1 pool that is retained at synapses even independently from F-actin after LTD stimuli. However, alternatively, it is also possible that the CaMKIIβ is sufficient for the complete suppression of DAPK1 re-accumulation.

### CaMKII synaptic accumulation by cLTP is unaffected by jasplakinolide

**Figure 4:**
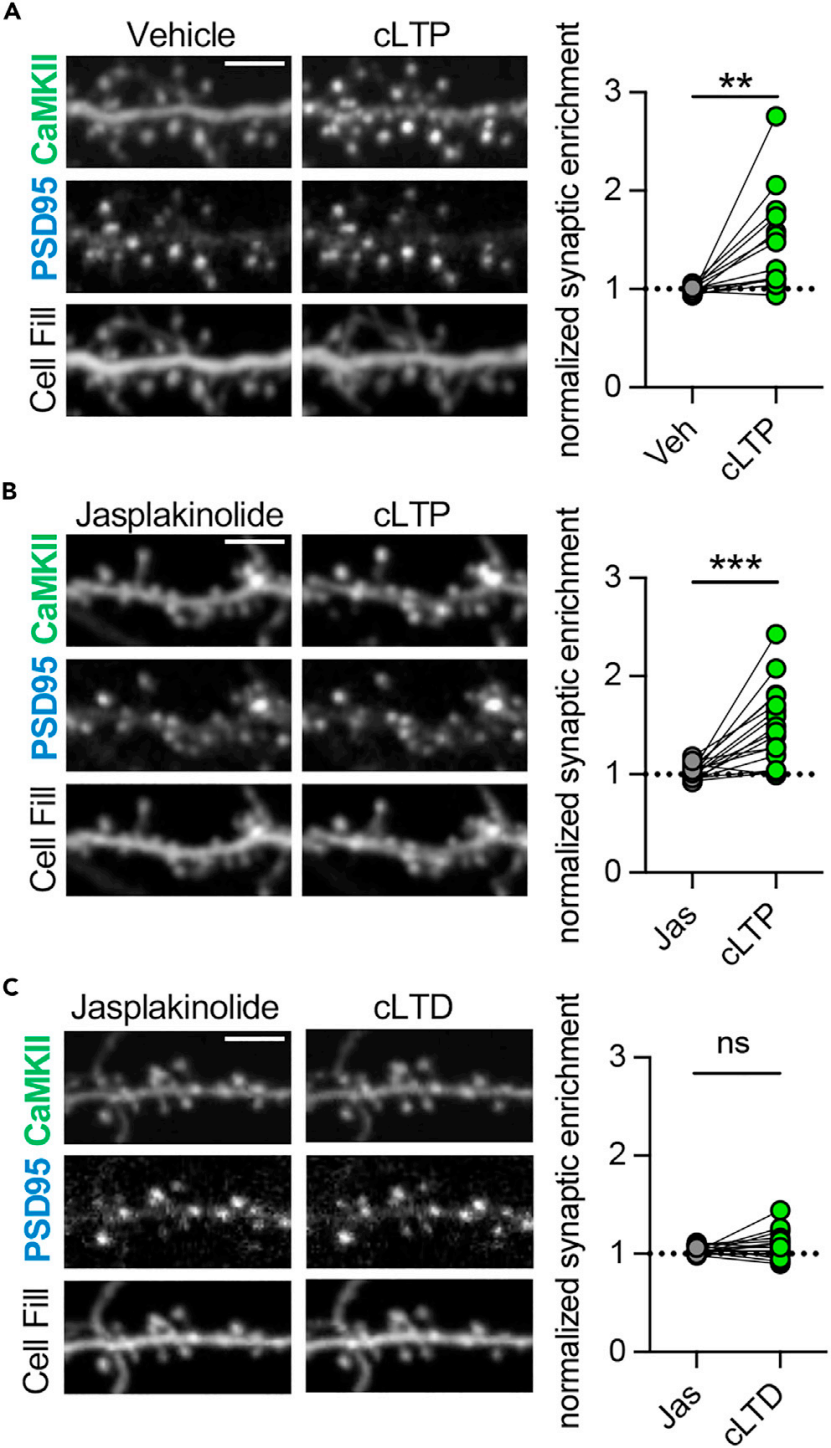
Stabilization of F-actin does not disrupt normal regulation of synaptic CaMKII accumulation Scale bars, 5 μm. (A and B) (A) cLTP stimulation results in the synaptic accumulation of CaMKII (n = 14 neurons; ∗∗p < 0.01; paired two-tailed t-test), which was unaffected by (B) jasplakinolide (n = 16 neurons; ∗∗∗p < 0.001; paired two-tailed t-test). (C) cLTD stimulation after pre-treatment with jasplakinolide does not result in CaMKII synaptic accumulation (n = 16 neurons; paired two-tailed t-test).

As destabilizing the F-actin cytoskeleton to disperse DAPK1 was sufficient to enable synaptic CaMKII accumulation even in response to cLTD stimuli, we next asked if stabilizing the F-actin cytoskeleton would vice versa be sufficient to suppress the CaMKII accumulation normally seen in response to cLTP stimuli (Figure 4A).^8^^,^^25^^,^^27^ As we had shown, stabilizing the F-actin cytoskeleton with jasplakinolide blocked synaptic removal of DAPK1 by cLTP stimuli (see Figures 1C and 1D). By contrast, for CaMKII, neither basal synaptic enrichment (Figure S4) nor further synaptic accumulation in response to cLTP stimuli (Figure 4B) was affected by jasplakinolide. Thus, while the synaptic F-actin-localized DAPK1 pool is necessary for the suppression of CaMKII movement in response to cLTD stimuli (see Figure 3C), it is not sufficient to suppress CaMKII movement in response to cLTP stimuli. This indicates that the F-actin-localized DAPK1 itself cannot suppress CaMKII movement, but that the F-actin localization is required to enable an LTD-specific transition to a DAPK1 pool that can compete with CaMKII for GluN2B binding.

### Jasplakinolide does not permit cLTD-induced CaMKII synaptic accumulation

As stabilizing F-actin did not disrupt the synaptic accumulation of CaMKII by cLTP, we then questioned whether jasplakinolide might enable cLTD stimuli to induce similar synaptic CaMKII accumulation. This could be the case, for instance, if the transient cLTD-induced F-actin depolarization is necessary for DAPK1 transition from an F-actin-bound pool to the F-actin-independent pool that suppresses CaMKII movement. Thus, we here measured CaMKII synaptic movement in response to cLTD stimuli after pre-treatment with jasplakinolide. However, stabilization of the F-actin cytoskeleton with jasplakinolide did not enable cLTD-specific synaptic CaMKII accumulation (Figure 4C). This indicates that F-actin destabilization, but not stabilization, prevents the formation of an F-actin-independent synaptic DAPK1 pool that suppresses synaptic CaMKII accumulation during LTD.

### The DAPK1 cytoskeleton binding domain is necessary but not sufficient for basal synaptic targeting

**Figure 5:**
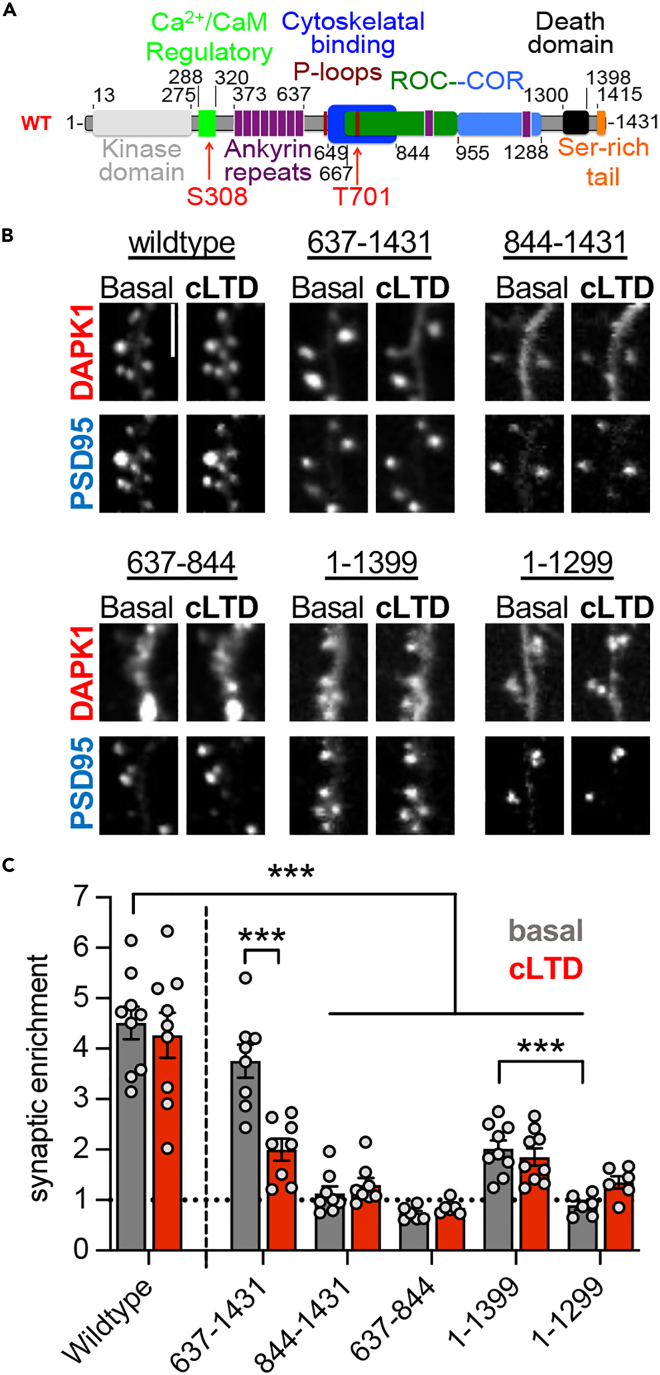
The DAPK1 cytoskeleton binding domain is necessary but not sufficient for basal synaptic targeting Scale bars, 5 μm. (A) DAPK1 structure and domains. (B) Representative images of mCh-DAPK1 truncation mutants before and after cLTD. (C) Synaptic enrichment values of mCh-DAPK1 truncation mutants basally (gray) and after cLTD (red) (∗∗∗p < 0.001; two-way ANOVA with Sidak’s multiple comparisons test). Individual data points are shown; the bar graphs represent mean ± SEM.

We next sought to determine which DAPK1 domains mediated synaptic localization. DAPK1 contains a known cytoskeletal binding domain (Figure 5A), which we postulated may be necessary and sufficient for basal DAPK1 synaptic localization. Indeed, an N-terminal deletion that extends just before the cytoskeletal binding domain (amino acids 637–1431) retained normal basal synaptic enrichment (Figures 5B and 5C). This fragment that lacks both the kinase domain and the ankyrin repeat region was not retained at synapses in response to cLTD, as expected from previous studies that showed that kinase activity of DAPK1 is required for the retention at synapses after cLTD stimuli.^7^ Also as expected, a further extended deletion of the cytoskeletal binding domain (amino acids 844–1431) completely abolished basal synaptic enrichment (Figures 5B and 5C), indicating requirement of the cytoskeletal binding domain for DAPK1 targeting to excitatory synapses. However, in contrast to our expectation, the cytoskeletal binding domain alone (amino acids 637–844) was not sufficient for basal synaptic localization (Figures 5B and 5C), indicating that an additional domain is required. Indeed, truncation of the C-terminal serine-rich tail while leaving the remainder of DAPK1 intact (amino acids 1–1399) dramatically impaired basal localization, but did not completely abolish it (Figures 5B and 5C). Further truncation that deleted the death domain (amino acids 1–1299) completely eliminated basal targeting (Figures 5B and 5C). Notably, the cytoskeletal binding domain alone (amino acids 637–844) showed clustering outside of dendritic spines (Figures 5B and S5), suggesting the possibility that this could be an indirect cause for the lack of spine localization. However, the C-terminal deletion constructs (amino acids 1–1399 and 1–1299) do not show such clustering (Figure 5B). Together, this indicates that the cytoskeletal binding domain, the death domain, and the serine-rich c-terminal tail are all required for normal DAPK1 basal synaptic localization, and that the cytoskeletal binding domain alone is not sufficient. Additionally, while the kinase domain is required for synaptic DAPK1 retention after LTD stimuli, it is not required for normal basal synaptic targeting. Together, these distinct domain requirements further support our proposed distinct mechanisms for basal synaptic DAPK1 targeting versus its synaptic retention after LTD.

## Discussion

DAPK1 has been thought to be targeted to dendritic spine synapses by its regulated binding to the NMDAR subunit GluN2B,^7^^,^^13^^,^^14^ and thereby control the synaptic localization of CaMKII that is mediated by competitive binding to GluN2B.^7^^,^^14^ Here, we show that basal synaptic DAPK1 localization instead requires F-actin. Notably, destabilizing F-actin caused a similar dispersal of DAPK1 from dendritic spines as seen after cLTP stimuli; vice versa, stabilizing F-actin prevented the cLTP-induced DAPK1 dispersal. This indicates that F-actin is required for the basal DAPK1 targeting to synapses and that the DAPK1 dispersal in response to cLTP stimuli requires the transient F-actin depolymerization. Notably, dendritic spine F-actin is disassembled after both LTP and LTD stimuli, although this disassembly is rather transient after LTP stimuli, but more persistent after LTD stimuli.^20^^,^^21^^,^^22^^,^^23^ In terms of the corresponding effects on CaMKII targeting, the removal of DAPK1 from synapses by destabilizing F-actin allowed the expected corresponding effects on synaptic CaMKII accumulation: after such DAPK1 removal, CaMKII accumulation at synapses was enabled even in response to cLTD stimuli. This is consistent with previous findings that showed synaptic CaMKII accumulation after cLTD stimuli is normally suppressed by synaptic DAPK1.^7^ However, maintaining DAPK1 at synapses even after cLTP stimuli by stabilizing F-actin did not have the corresponding opposite effect on CaMKII: CaMKII still accumulated at synapses after such cLTP stimuli. This indicates that DAPK1 binding to F-actin is necessary but not sufficient to suppress synaptic CaMKII movement. This led us to our model in which CaMKII movement is suppressed by DAPK1 via competitive binding to GluN2B, as originally proposed.^7^ However, the induction of this competitive DAPK1 binding to GluN2B occurs only in response to cLTD stimuli and requires an initial basal synaptic targeting of DAPK1 that is mediated by the F-actin binding instead. Indeed, after cLTD stimuli, synaptic localization of DAPK1 was no longer strictly dependent on F-actin; vice versa, after dispersing DAPK1 from synapses by depolymerizing F-actin, cLTD stimuli were not sufficient to allow synaptic re-accumulation of DAPK1 but instead allowed synaptic accumulation of CaMKII.

Overall, our results indicate two distinct but connected pools of synaptic DAPK1, one that is localized via F-actin and another that is localized via GluN2B. The F-actin-dependent pool mediates basal synaptic DAPK1 localization whereas the other pool mediates synaptic DAPK1 retention after cLTD stimuli. The pools are interconnected, because even though the F-actin-dependent pool is not sufficient to suppress CaMKII accumulation, it is necessary for the cLTD-induced generation of the other pool of DAPK1 that does suppress CaMKII accumulation after cLTD stimuli. The F-actin-independent pool for DAPK1 is likely bound to GluN2B to suppress CaMKII binding, but may still additionally remain directly F-actin-bound, even if this binding is no longer strictly necessary for its localization. Notably, for CaMKII, we here studied the major brain isoform, CaMKIIα; in contrast to another brain isoform, CaMKIIβ, this isoform does not show much direct F-actin binding.^28^^,^^29^^,^^30^

In contrast to our findings here, a previous report demonstrated that stabilizing F-actin using jasplakinolide was sufficient to restrict the synaptic CaMKII movement that is induced by LTP stimuli.^31^ However, this former study utilized long-term applications (3+ hours) of jasplakinolide that have been shown to induce the formation of amorphous actin structures that can plug the spine neck to restrict access of even smaller molecules.^31^^,^^32^ By contrast, the short-term jasplakinolide treatment (1 μM, 10 min) utilized in our study still appears to allow for molecular diffusion into and out of the spine. Indeed, whereas jasplakinolide disrupted the removal of DAPK1 from synapses, it still allowed CaMKII synaptic accumulation by cLTP. These data indicate: 1) short-term jasplakinolide treatment does not restrict general entry or exit of molecules in dendritic spines, 2) DAPK1 and CaMKII synaptic localization are not mutually exclusive, and 3) the F-actin-localized DAPK1 pool is not sufficient to mediate suppression of CaMKII movement, but is required for initiation of DAPK1 localization to the LTD-specific, GluN2B-bound pool.

It should be noted that the results of our experiments do not demonstrate a direct interaction between DAPK1 and F-actin. While our results demonstrate that F-actin is required for the synaptic targeting of DAPK1 under basal conditions, this does not rule out the possibility that this is mediated by additional intermediate binding partners. Furthermore, while F-actin is required, our results suggest that F-actin binding is not sufficient for DAPK1 targeting to synapses. Similar as previously shown for F-actin co-localization of DAPK1 in other cells,^33^ synaptic DAPK1 localization in hippocampal neurons was abolished by deleting the cytoskeleton binding domain but not by deleting the ankyrin repeats. However, unlike association with the cytoskeleton in other cells,^34^ the cytoskeleton binding domain alone was not sufficient for synaptic targeting; instead, the C-terminal Ser-rich region and death domain appeared to be required in addition.

Consistent with our model of LTD-induced reorganization of the synaptic binding modes of DAPK1, our results showed that the DAPK1 domain requirement differs for basal synaptic targeting versus synaptic retention after LTD stimuli: While a C-terminal fragment that includes the cytoskeletal binding domain is sufficient for basal targeting, the N-terminal kinase domain is additionally required for the retention. The most likely additional interaction that is induced by LTD stimuli is with the NMDAR. Indeed, this interaction was demonstrated to occur *in vitro* and was originally erroneously thought to mediate also the basal synaptic targeting of DAPK1.^7^^,^^13^ Moreover, DAPK1 versus CaMKII binding to the NMDAR is competitive,^7^^,^^14^ which can directly explain the cross-regulation between the two kinases. However, verification of this plausible assumption in future studies will require additional tools, such as NMDAR mutants that are impaired for DAPK1 binding.

### Limitations of the study

Our study strongly suggests that the two pools of DAPK1 identified here are characterized by their binding to F-actin versus GluN2B, respectively. However, one of the pools may involve binding to both proteins. Also, although GluN2B is the most likely candidate for binding of one of the pools (as this would directly cause the DAPK1 effect on CaMKII localization), the persistent DAPK1 localization after LTD stimuli could instead be mediated by another synaptic protein. Furthermore, while we demonstrated that F-actin is required for the localization of the other synaptic DAPK1 pool, this could be mediated by an indirect interaction rather than by direct binding.

## STAR★Methods

### Key resources table

**Table:** 

REAGENT or RESOURCE	SOURCE	IDENTIFIER
**Chemicals, peptides, and recombinant proteins**		

Papain	Worthington	LS 03126
Lipofectamine 2000	Invitrogen	11668027
B-27 supplement	Gibco	17504044
Penicillin-Streptomycin	Gibco	15070063
Neurobasal-A Medium	Gibco	10888022
MEM	Gibco	11090081
5-Fluoro-2′-deoxyuridine (FdU)	Sigma	F0503
Glutamate	Sigma	6106-04-3
Glycine	Sigma	56-40-6
CNQX	Sigma	115066-14-3
NMDA	Sigma	M3262
Latrunculin B	Enzo Life Sciences	BML-T110-0001
Jasplakinolide	Adipogen Corporation	AG-CN2-0037-C100

**Recombinant DNA**		

mCherry-DAPK1	Goodell et al.^7^	Clonetech #632524
mCherry-CaMKIIα	In house	N/A
mTurquois-PSD95 intrabody	Gross et al.^16^; Cook et al.^15^	Addgene #46295
mCitrine	Addgene	Addgene #54594

**Experimental models: Cell lines**		

Primary hippocampal cultures	Laboratory of K. Ulrich Bayer	N/A

**Experimental models: Organisms/strains**		

Mouse: CaMKIIα knockout	In house	N/A
Rat: Sprague-Dawley	Charles River Labs	N/A

**Deposited data**		

Raw and analyzed data	This paper	https://data.mendeley.com/datasets/37xjryhnf6/1

**Software and algorithms**		

Slidebook 6.0	Intelligent Innovations (3i)	RRID:SCR_014300
Prism 7.0	Graphpad	RRID: SCR_002798
ImageJ	NIH	RRID:SCR_003070

### Resource availability

#### Lead contact

Further information and requests for resources and reagents should be directed to and will be fulfilled by the Lead Contact, K. Ulrich Bayer (ulli.bayer@ucdenver.edu).

#### Materials availability

Plasmids used in this work will be available upon request.

### Experimental model and subject details

All animal treatment was approved by the University of Colorado Institutional Animal Care and Use Committee, in accordance with NIH guidelines. Animals are housed at the Animal Resource Center at the University of Colorado Anschutz Medical Campus (Aurora, CO) and are regularly monitored with respect to general health, cage changes, and overcrowding. Pregnant Sprague-Dawley rats were supplied by Charles River Labs. Mice were bread in house. Hippocampal cultures were prepared on postnatal day P0 (rat) or P1 (mouse) and imaged on day *in vitro* (DIV) 15–18.

### Method details

#### Material and DNA constructs

Material was obtained from Sigma, unless noted otherwise. The mTurquois-labelled intrabody^15^ does not affect synaptic functions.^16^ Expression of the intrabody is driven by the CAG promoter; expression of all other constructs is driven by the CMV promoter. The N-terminal DAPK1 truncation mutants were generated via PCR amplification of the indicated amino acid regions and insertion into mCherry-C1 vector using the Kpnl and Apal sites. C-terminal truncations were made via insertion of STOP codons using site-directed mutagenesis. The construct for expression of mCherry-CaMKII was made based on an mGFP-CaMKIIα^27^ construct by switching the mGFP for an mCherry using the Eco47III and BsrgI sites. All constructs were validated by sequencing.

#### Primers

The following primers were used to make C-terminally truncated DAPK1 by stop codon insertion: (1–1299) GGCCAGCCTCGGCATGGACTGACATGCATCAGACCTGAACC; GGTTCAGGTCTGATGCATGTCAGTCCATGCCGAGGCTGGCC;

(1–1399) GGCATCCTCTGTGTTCTAAATCAACCTGGATGGC; GCCATCCAGGTTGATTTAGAACACAGAGGATGCC.

The following primers were used to make N-terminally truncated DAPK1 by Gibson assembly: (637–844) CAAGCTTCGAATTCTGCAGTCGACGGTACCGAGGCGCTGACCACGGACGGAAAGAC; GTTATCTAGATCCGGTGGATCCCGGGCCCTCACTCTTCTAGACTAAAGACAACAACA;

(637–1431) CAAGCTTCGAATTCTGCAGTCGACGGTACCGAGGCGCTGACCACGGACGGAAAGAC; AGTTATCTAGATCCGGTGGATCCCGGGCCCTCACCGGGATACAACAGAGCTAATGGA
(844–1431) CAAGCTTCGAATTCTGCAGTCGACGGTACCGAGCCCTATGAGATCCAGCTGAACCAA; AGTTATCTAGATCCGGTGGATCCCGGGCCCTCACCGGGATACAACAGAGCTAATGGA.

#### Primary hippocampal culture preparation

To prepare primary hippocampal neurons, hippocampi were dissected from mixed sex rat pups (P0), dissociated in papain for 1 h, and plated at 100,000 cells/mL on 18 mm No. 1 deckgläser cover glasses in plating media (MEM containing 10% FBS, 1% Penicillin-Streptomycin). For CaMKIIα KO cultures, hippocampi were dissected from mixed sex mouse pups (P1), dissociated in papain for 30 minutes, and plated at 250,000 cell/mL. For all cultures, on DIV 1 plating media was replaced with feeding media (Neurobasal A containing 2% B27 and 1% Glutamax). On DIV 7, half of conditioned feeding media was replaced with fresh feeding media containing 2% 5-Fluoro-2′-deoxyuridine (FdU).

#### Image acquisition and analysis

Neurons were imaged using a Axio Observer microscope (Carl Zeiss) fitted with a 63× Plan-Apo/1.4 numerical aperture (NA) objective, 50 μm pinhole, using 445, 515, and 567 nm laser excitation and a CSU-XI spinning disk confocal scan head (Yokogawa) coupled to an Evolve 512 EM-CCD camera (Photometrics) and controlled using Slidebook 6.0 software (Intelligent Imaging Innovations [3i]. The 63× immersion objective (4.92 pixels/micron) was used to acquire all images.

DIV 15–18 rat neuronal cultures were transfected with Lipofectamine 2000 (Invitrogen) to express mTq-PSD-95 intrabody, mCitrine cell fill, and mCh-CaMKII or mCh-DAPK1 (WT or noted mutations) and imaged 24 hours after. Images were collected at 32°C in HEPES buffered imaging solution (ACSF) containing (in mM) 130 NaCl, 5 KCl, 10 HEPES pH 7.4, 20 Glucose, 2 CaCl_2_, 1 MgCl_2_. Images of individual neurons from at least two independent cultures were acquired by 0.5 μm steps over 6 μm. 2D maximum intensity projection images were then generated and analyzed using ImageJ. Regions of dendrites were masked in two-ways. Threshold for entire cell was manually defined using the mCitrine fluorescence to include both dendritic spines and the dendritic shaft. Threshold for excitatory synapses was manually defined using mTurquois-PSD-95 fluorescence to label only synapses. To create a mask that includes only dendritic shaft while omitting any excitatory, PSD-95-containing synaptic regions, the excitatory synapse mask was dilated and subtracted from the mCitrine cell fill mask. mCh-CaMKII or mCh-DAPK1, mTq-PSD95 and mCitrine synaptic enrichment were measured using the ratio of average fluorescence intensity within the synapse mask to the average fluorescence intensity within the shaft mask.

#### Latrunculin B and jasplakinolide treatment

Latrunculin B (2.5 μM, DMSO 0.01% v/v in ACSF) was applied for 5 minutes prior to imaging and washout. Jasplakinolide (1 μM, DMSO 0.01% v/v in ACSF) was applied for 10 minutes prior to imaging and washout.

#### Chemical LTD and LTP stimulation

In the imaging chamber, chemical LTD (cLTD) was induced with 30 μM NMDA, 10 μM glycine, and 10 μM CNQX for 1 min. Chemical LTP (cLTP) was induced with 100 μM glutamate and 10 μM glycine for 45 seconds. Both treatments were followed by washout with 5 volumes (5 mL, 1mL/10sec) of fresh ACSF. When applicable (see legends), latrunculin or jasplakinolide were included in the ACSF washout.

### Quantification and statistical analysis

All data are shown as mean ± SEM. Statistical significance is indicated in the figure legends. Statistics were performed using Prism (GraphPad) software. Imaging experiments were obtained and analyzed using SlideBook 6.0 software. All comparisons between two groups met parametric criteria, and independent samples were analyzed using unpaired, two-tailed Student’s t-tests. Comparisons between three or more groups meeting parametric criteria were done by one-way ANOVA with specific post-hoc analysis indicated in figure legends. Comparisons between three or more groups with two independent variables were assessed by two-way ANOVA with Bonferroni post-hoc test to determine whether there is an interaction and/or main effect between the variables. Asterisks represent level of significance: ∗p < 0.05; ∗∗p < 0.01; ∗∗∗p < 0.001.

## Data Availability

• Data: The datasets generated during this study are available through Mendeley (https://data.mendeley.com/datasets/37xjryhnf6/1). • Code: This paper does not generate original code. • Other itmes: not applicable.
