# Supplementary material for: Distinct synaptic pools of DAPK1 differentially regulate activity-dependent synaptic CaMKII accumulation
Source: iScience. 2023 Apr 23;26(5):106723. doi: 10.1016/j.isci.2023.106723 (PMC10192646; doi:10.1016/j.isci.2023.106723)
Supplement: Document S1. Figures S1–S5 [file mmc1.pdf]

iScience, Volume *26*

Supplemental information

Distinct synaptic pools of DAPK1 differentially regulate activity-dependent synaptic CaMKII accumulation

Jonathan E. Tullis and K. Ulrich Bayer

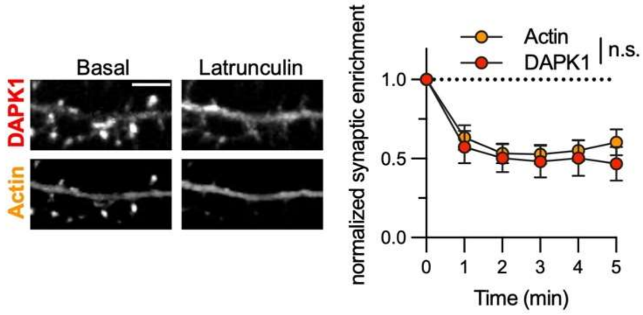

Supplemental Figure S1. Acute application of latrunculin reduces actin and DAPK1 synaptic localization equally, related to Figure 1.

Representative images of mCh-DAPK1 and YFP-actin (left); Scale bars, 5 µm. Quantifications (right) show mean ± SEM. Synaptic enrichment values were measured over time following treatment with Latrunculin B (2.5 µM) for 5 minutes (n = 3 neurons; two-way RM ANOVA with Tukey's multiple comparison's test; time: p<0.001; group: not significant, n.s.).

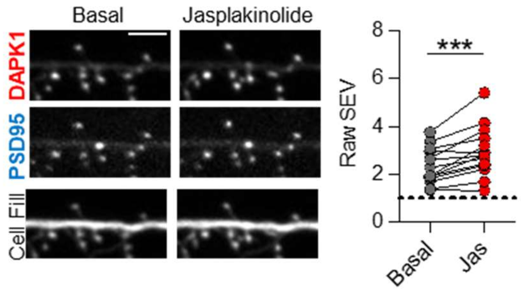

Supplemental Figure S2. Acute application of jasplakinolide enhances DAPK1 synaptic localization, related to Figure 1.

Quantifications show mean ± SEM. Scale bars, 5 µm. Stabilization of F-actin with jasplakinolide (1µM, 10 min) increased DAPK1 synaptic enrichment (n = 14 neurons; ***p<0.001 in paired two-tailed t-test).

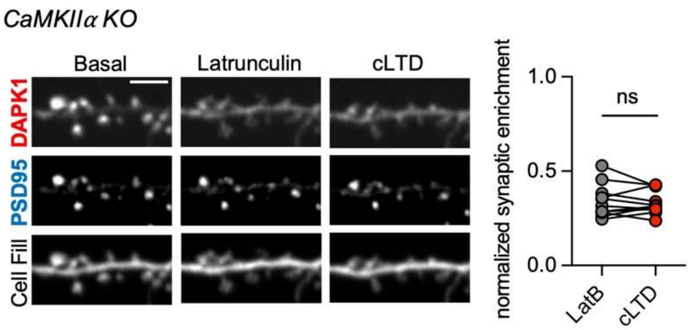

Supplemental Figure S3. CaMKII KO does not enable LTD-induced re-accumulation of DAPK1 after synaptic removal, related to Figure 2.

Scale bars, 5 μm. DAPK1 does not re-accumulate to synapses by cLTD after synaptic removal by latrunculin in CaMKII KO cultures (n = 11 neurons; not significant, ns, in paired two-tailed t-test).

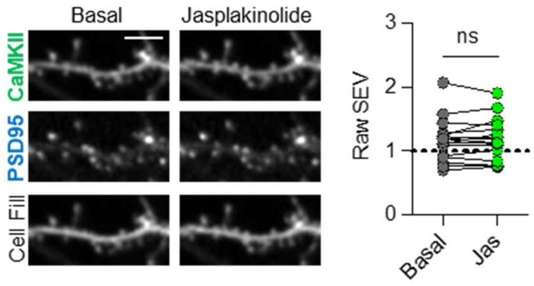

Supplemental Figure S4. Acute application of latrunculin does not affect basal CaMKII synaptic localization, related to Figure 4.

Scale bars, 5 μm. Stabilization of F-actin with jasplakinolide (1μM, 10 min) did not affect CaMKII synaptic enrichment (n = 16 neurons; not significant, ns, in paired two-tailed t-test).

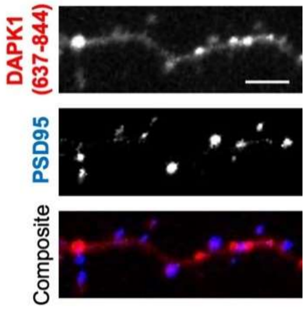

Supplemental Figure S5. Extrasynaptic clustering of DAPK1 aa. 637-844, related to Figure 5.

Scale bar, 5 μm. Representative images of DAPK1 (red, aa. 637-844) and PSD-95 intrabody (blue). The DAPK1 cytoskeletal binding domain alone formed extrasynaptic clusters which were distinctly separated from PSD-95 puncta.
